# Dental treatment under general anesthesia in mentally disabled patients based on an ambulatory surgery model: A Case-control study

**DOI:** 10.4317/jced.59266

**Published:** 2022-02-01

**Authors:** Cecilia-Fabiana Márquez-Arrico, Julio Talaván-Serna, Francisco-Javier Silvestre, Juan Viñoles, Sandra Rodríguez-Martínez, Javier Silvestre-Rangil

**Affiliations:** 1 Stomatology Service, Hospital Universitario Dr. Peset-FISABIO; 2 Anesthesiology Unit, Obispo Polanco Hospital, Teruel, Spain; 3 Department of Stomatology, University of Valencia, Spain; 4 Anesthesiology Unit, University Hospital Dr. Peset, Valencia, Spain; 5 Emergency Unit, Obispo Polanco Hospital, Teruel, Spain

## Abstract

**Background:**

Mentally disabled patients commonly offer little or no cooperation in dental treatments, and general anesthesia may become necessary in such cases. The present study was to identify the most relevant factors in dental treatment under general anesthesia in disabled patients based on a Major Ambulatory Surgery (MAS) model. The study analyzes anesthetic variables and type of dental procedures carried out for disabled patients compared with controls.

**Material and Methods:**

A case-control study was carried out with 574 patients (263 cases and 311 controls) subjected to dental treatment under general anesthesia in the Day Surgery Unit of Dr. Peset University Hospital (Valencia, Spain). Epidemiological, anthropometric and preoperative data (ASA score, Mallampati classification) were collected.

**Results:**

Males and obesity were more prevalent among disabled patients than controls. Significant associations were found between longer surgery time, underwent thoot extraction, tartrectomy, fillings and disabled patients treated under general anesthesia. The preoperative risk scores were likewise higher in disabled patients (ASA III-IV). The duration of surgery increased with the ASA score but didn´t influence postoperative stay. Patient condition in the first 24 hours of late postoperative recovery was good in both groups.

**Conclusions:**

Dental treatment based on the MAS in mentally disabled patients is effective and safe, even in individuals with a certain prior risk (ASA III).

** Key words:**Disabled patients, ambulatory surgery, dental treatment, special needs, Major Ambulatory Surgery by general anesthesia.

## Introduction

Dental treatment on disabled patients is often difficult because of their behavior (depending on collaboration) general health status, the medication used to treat their pathologies, and social condition (1). The World Health Organization (WHO) proposes the following definition of disabilities: is an umbrella term, covering impairments, activity limitations, and participation restrictions Disability is thus not just a health problem, but a complex phenomenon, reflecting the interaction between features of a person’s body and features of the society in which he or she lives (2,3).

Patients with functional problems may present different degrees of mental impairment that pose a challenge for dental treatment (3) . On the other hand, deficiencies and limitations of a medical, economical, social, and behavioural nature complicate the access of these patients to conventional dental care, and special clinical management measures may be required, including the correction of behaviour, physical stabilization or premedication in order to perform the dental treatment (3).

Oral problems affect practically all individuals with mental disabilities - the prevalence being clearly higher than that found in the general population (4). Disabled patients can present a higher incidence of disorders such as caries (4) and gingivitis or periodontitis (1,5-7), due to a lack of cooperation and deficient dental hygiene which often needs to be performed on an assisted basis. The presence of malocclusions is also frequent (5). Disabled patients frequently present dental trauma, drooling and negative habits such as bruxism (8). In some cases, self-injuring practices such as chronic nibbling can be seen, resulting in important oral mucosal lesions (8). In relation to dental treatment, mentally disabled patients commonly offer little or no cooperation, and general anesthesia in the operating room may become necessary in these cases (9).

Dental procedures are a frequently reason for general anesthesia in children. These procedures vary in duration from a few minutes for removal of a tooth, to a few hours for dental restoration procedures. Guidelines limit the degree of sedation during treatment in the dental chair, and complex work in anxious or young children is performed in a hospital under general anesthesia. Major Ambulatory Surgery (MAS) programs in dental care have afforded great benefits for patients with special needs, children, and particularly for patients with severe mental disabilities since treatment can be carried out under general anesthesia or deep sedation. In the specific case of patients with severe mental disabilities, dealing with them outside their usual surroundings can be problematic because of their behavioral disorders, since hospital wards are usually not prepared to receive such patients. Nevertheless, treatment under general anesthesia and in the hospital setting affords greater safety and efficacy, resulting in greater quality patient care (10). To sum up, very few studies have been published on the procedures and protocols required for dental treatment under general anesthesia among patients with special needs (11,12). Thus, the aim of this study was to identify the most relevant factors in dental treatment under general anesthesia in disabled patients based on a MAS model.

## Material and Methods

-Study Sample

A case-control study was carried out with 574 patients males and females patients aged between 3 and 89 years old subjected to dental treatment under general anesthesia in the Day Surgery Unit of Dr. Peset University Hospital (Valencia, Spain) in the period between 2014-2018. The cases group consisted of 263 mentally disabled patients in which handling problems precluded conventional treatment under local anesthesia with correction of behavior, physical restraints or premedication. The control group consisted of 311 patients without disabilities that were seen in the Stomatology Outpatient Clinic due to impacted third molars, cysts or lesions requiring surgical treatment under general anesthesia. Likewise, there were some patients with intense preoperative anxiety requiring general anesthesia or deep sedation for correct dental treatment (Table 1).

**Table 1:**
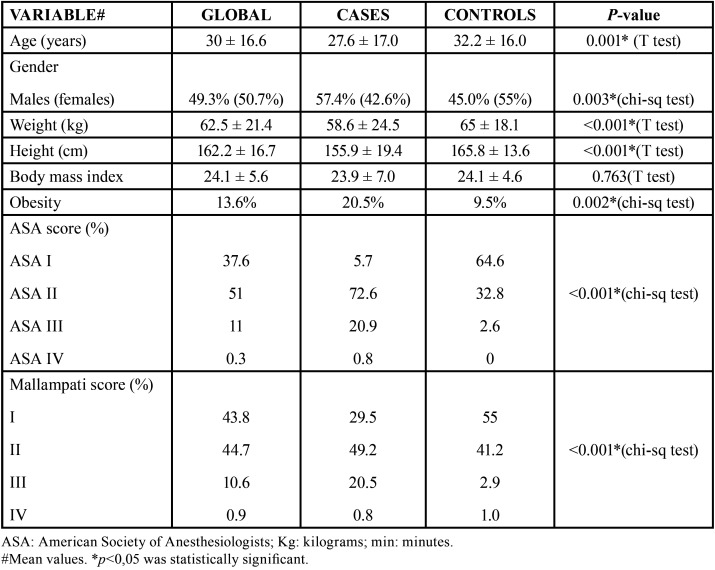
Characteristics of Study Sample.

-Study Design

These subjects were referred to the Stomatology Unit of the hospital from two dental clinics specialized in the care of patients of this kind (Red Cross dental clinic for special needs patients in Valencia, and dental clinic of the University of Valencia). The hospital Ethics Committee approved the study. Epidemiological data such as age and gender were recorded of a patient’s clinical data and medical history, along with anthropometric parameters such as weight, height, body mass index (BMI), and the presence of overweight and obesity.

Pre-anesthesia assessment included a good history, a physical examination performed by the anesthesiologist (electrocardiogram, radiographs, consultations) and any indicated laboratory tests. Preoperative data were compiled, including anesthesia risk according to the American Society of Anesthesiologists (ASA) score and the Mallampati classification for preoperative airway assessment. The types of treatment were recorded, i.e., tartrectomy, fillings, extractions and oral surgery (impacted teeth, cysts, etc.), and we documented the number of extractions per patient, as well as the number of fillings. The data were recorded in medical histories by the doctors who perform the interventions 

The appearance of post-treatment complications was evaluated, along with the “postoperative indicator” – a telephone survey score systematically used by the Day Surgery Unit (DSU) of our hospital to assess patient condition in the first 24 hours of late postoperative recovery at home (13). The indicator is applied to all types of surgery in the DSU, and is based on the simple sum of four specific items: bleeding, general condition, pain, and tolerance. The items are scored ranging from negative values in the case of poor patient evolution (need for medical care) to values between 4 and 8 reflectings a normal postoperative course. Surgery time and the duration of stay in the DSU were also analyzed.

-Statistical Analysis

Continuous variables were reported as the mean and standard deviation (SD), and categorical variables as absolute frequencies and percentages. The Student t-test for independent samples was used to assess homogeneity between the groups (means of cases and controls), while the chi-squared test was used to measure the degree of dependency between categorical variables, and the Fisher exact test was applied for 2 x 2 comparisons. Analysis of variance (ANOVA) was used to contrast means referred to surgery time and duration of stay in the DSU, according to the ASA score and adjusted for age and gender. Multiple comparisons were made using the Bonferroni correction. Multiple linear regression analysis was used to explore the relationship between the postoperative indicator scores and the surgical and postoperative times adjusted for age and gender. Binary logistic regression analysis, in turn, estimated the odds ratios (ORs) of the associations of the different factors. The level of statistical significance was established as 5% (α=0.05). The SPSS version 19.0 (SPSS Statistics Inc., Chicago, IL, USA) was used throughout.

## Results

-Characteristics of Study Sample

Descriptive statistics showed that the controls were older (*p*=0,001) on average than the disabled patients (32.2±16.0 vs. 27.6±17.0 years, respectively). However, there were comparatively more patients under 20 years old in the group of disabled patients, and more patients between 21-29 years old range than in the control group (40.3% versus 30%). There was a greater presence of females in the control group (55% versus 42.6%; *p*=0.003). A slight male predominance was observed in the global cohort (50.7%; *p*=0,003) (Table 1).

With regard to the anthropometric parameters, mean body weight was greater in the control group (65.6±18.1 versus 58.6±24.5 kg; *p*<0.001), in the same way as body height (165.8±13.6 versus 155.9±19.4 cm; *p*<0.001). However, no differences were observed between the groups in terms of BMI (global mean 24.1±5.6 kg/m2). Obesity was globally more prevalent in disabled patients (20.5% versus 9.5%; *p*=0.002). However, these patients were characterized by a great variability of BMI – some individuals presenting very low BMI and others very high values.

-Results of Anesthetic Variables

In relation to the preoperative ASA score, 64.6% of the controls were ASA I and 32.8% ASA II. In comparison, the disabled patients were more widely distributed among ASA I (5.7%), ASA II (72.6%), ASA III (20.9%) and ASA IV (0.8%) – with clear differences between the groups (*p*<0.001). The Mallampati score in the group of disabled patients was higher than in the control group, with greater difficulty for securing airway control (Table 1).

**Figure 1:**
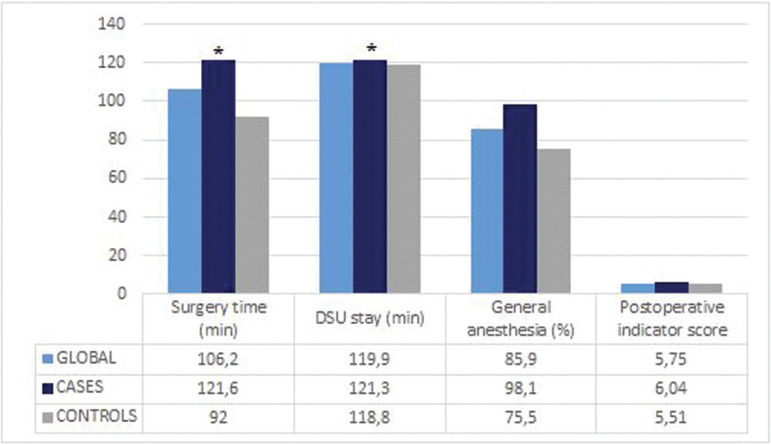
Anesthetic variables. *p<0,001 T test was used to compare surgery time and DSU stay between cases and controls. Chi-sq test was used to compare postoperative indicator score and DSU: Day Surgery Unit between cases and controls.

General anesthesia was the anesthetic technique most frequently used in disabled patients (98.1% versus 75.6% in the control group, *p*<0.001). One-third of the controls were subjected to monitored anesthetic care with intravenous sedation and local anesthesia (Fig. 1). The mean surgery time in the control group was 92.0±38.7 min, and was significantly longer among the disabled patients (121.6±43.7 min, *p*<0.001) – this was reflecting the need for more time to complete treatment in patients with mental disabilities. However, the mean postoperative stay in the DSU was 118.8±48.3 min among the controls and similar in the disabled patients (121.3±49.6 min, *p*=0,570). The relationship between surgery time and the duration of admission to the DSU showed abriefer surgery being associated with a shorter stay in the DSU (Fig. 1).

The surgery time in the global sample was seen to increase with the ASA score (*p*<0.001). Specifically, surgery time increased 25 min between ASA I and ASA II, and also between ASA II and the patients with ASA III and IV. However, considering the duration of stay in the DSU, the values were more homogeneous among the different ASA scores, i.e., the latter did not influence the duration of stay in the DSU (*p*=0.845) (Fig. 1)

The postoperative indicator could not be established in 1.4% of the global series (evenly distributed between the two groups), because telephone contact with the patient or caregivers did not prove possible. In relation to this indicator, it must be noted that the general condition score was much lower among the controls than in the mentally disabled patients. Only in the case of pain were the scores seen to be significantly lower among the disabled patients than in the controls (*p*=0,001).

The mean score corresponding to the four items of the postoperative indicator was 5.75±1.88 in the global sample, 5.51±1.92 in the controls, and 6.04±1.80 among the disabled patients. In the global study sample, negative postoperative indicator scores were only recorded on two occasions, corresponding to patients who return to hospital after having been discharged home. In one case the reason was to be in pain (with patient discharge home from the emergency room) and in the other was post-extraction bleeding (with admission to hospital).

-Results of Dental Procedures Carried Out

**Figure 2:**
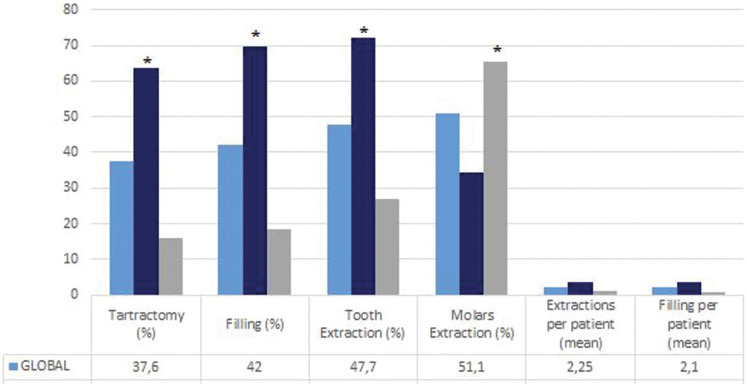
Dental procedures carried out. *p<0,001. Chi-sq test was used to compare the porcentage of tartrectomy, filling, tooth extraction and molars extraction carried out between cases and controls. T test was used to compare the number of extractions per patient and filling per patient between cases and controls.

Dental cleaning with ultrasound was carried out in 63.6% of the disabled patients. Fillings were also very commonly performed in these subjects (69.6%), in contrast to the control group (*p*=0,001), where practically no fillings were made. The mean number of fillings per patient in the cases group was 3.53 while for patients in the control group was 0,9 (Fig. 2).

The prevalence of simple extractions (including third molars) was 27% in the control group and 72.2% among the disabled patients (*p*=0,001). The number of extractions per patient was 2.25±3.95 for the global cohort (1.15 in the control group and 3.56 in the disabled patients, *p*=0,001). Overall, third molar surgery accounted for over half of all treatments (51.1%), though it proved significantly more frequent in the control group (65.3%) than among the disabled patients (34.2%, *p*<0.001). In the control group, molar extractions were more frequent (65,3% control and 34,4% cases, *p*=0,001) in individuals with lower ASA scores and in the 30-39 years old age range. The frequency of tartrectomy was seen to decrease as patient age increased, and was found to be greater among younger individuals and in those with higher ASA scores. In relation to the types of treatment and the ASA score, the number of extractions was seen to increase with age and was higher in patients with ASA III and IV than in those with ASA II. The number of fillings also increased with age, though up until 40 years old of age the ASA I patients required fewer fillings than those with higher ASA scores (*p*<0.001).

## Discussion

The present study compared two cohorts of patients (mentally disabled patients and controls) referred for dental treatment in a DSU, based on a MAS model. Although the two groups were comparable, the controls programmed for oral surgery were somewhat older than the disabled patients and had a greater prevalence of females. With regard to the anthropometric variables, no differences were observed between the two groups in terms of BMI, though important variability was observed among the disabled patients – with both obese individuals and patients with very low BMI values.

The evaluation of anesthetic risk based on the ASA score showed the disabled patients to have a clearly greater risk than the controls, in concordance with the observations of other authors (13). However, and in coincidence with other studies, this increased anesthetic risk did not cause any patient to be excluded from treatment under general anesthesia (14).

All of the patients with mental disabilities were treated under general anesthesia due to deficient cooperation and the clinical management problems they posed. In contrast, one-third of the controls were treated under intravenous sedation and with local anesthesia, since the management of these patients was clearly easier. The disabled individuals were referred from two specialized clinics for patients of this kind. Only one-third of the patients seen in these clinics were referred to our center for treatment under general anesthesia, being this proportion very low in comparison with data found in the literature (15,16). A possible explanation for this is that such patients had already been previously evaluated in the mentioned clinics by dentists specialized in cases of this kind, and most of those who were not referred to our center could be treated following correction of behavior, physical restraints or premedication. In contrast, patients who were referred presented cognitive disturbances, severe mental retardation or aggressive behavior, in line with the observations of other investigators (17-19). A frequent problem with these patients referred for treatment under general anesthesia is that it is not possible to perform prior oral explorations or complementary tests such as panoramic X-rays, due to the important management problems they pose. In order to do a preoperative planification, it could be interesting to carry out a dental check up using conscious sedation with midazolam.

On the other hand, controls were referred from the hospital outpatient clinic for procedures such as the extraction of impacted teeth, maxillary cyst removal or oral soft tissue surgery. Most of the disabled patients were receiving dental treatment for the first time (20), having been selected for general anesthesia due to the impossibility of using other treatment techniques on an ambulatory basis. In almost two-thirds of the cases, the treatments required by the disabled patients were extractions and fillings due to caries being consistent with the observations of other authors (9-21). A lesser proportion required dental cleaning or nonsurgical periodontal treatment. In this subgroup, only one-third required molar extractions.

It must be taken into account that in the case of doubt as to whether restorative treatment might fail, direct extraction was decided in mentally disabled patients because of the complexity and risk of complications associated with possible retreatment procedures under general anesthesia in these individuals (22). In some studies, 10% of the patients treated under general anesthesia required a second operation under general anesthesia (9). Some authors have even reported that 3% of the patients required a third treatment under general anesthesia in the course of follow-up (12). In general, the retreatment rate in the 5 years following the first treatment under general anesthesia is estimated to be 4-12%. However, in order to preserve the effects of initial treatment and avoid new oral lesions, these patients would have to be enrolled in a post-treatment prevention program with full implication on the part of the caregivers (12,20). In any case, the ideal strategy would be to adopt early prophylactic measures with the purpose of avoiding the number of extractions required by these patients in a later stage (14). Although different classifications of the types of treatments used and their duration have been proposed (12,22), most of them divide the patients into two groups: those requiring only extractions under general anesthesia, and those requiring fundamentally dental prophylaxis, fillings, and extractions. This has been the approach adopted in our study. Other types of dental procedures, such as root canal treatments in severely degraded posterior teeth, greatly complicate the timing and duration of treatment under general anesthesia and may result in restoration failure over the long term. Nevertheless, some authors perform endodontic treatments and restorations in a single step (23), as well as the placement of implants, with good results (24,25). Obviously, procedures involving the fitting of prostheses or orthodontics fall outside this scenario of treatment under general anesthesia.

The surgery times were longer in the disabled patients than in the control group due to two reasons: (a) the complexity of management of uncooperative disabled patients prolonged the anesthetic induction and eduction times; and (b) the need for combined surgical and dental procedures in one same surgical session (dental prophylaxis, fillings, and extractions or surgery). Nevertheless, the time to discharge home was similar in both groups. A possible explanation of this finding could be that the public health system offers surgical procedures for all patients (disabled and non-disabled) but not restorative and preventive treatment for non-disabled patients. Following premedication in the form of oral midazolam, induction was carried out with inhaled anesthetics, and anesthesia was then completed via the intravenous route, with nasotracheal intubation and pharyngeal block using dressing impregnated in saline solution. Although nasotracheal intubation was used in most patients, in those cases in which the tube could not be advanced through the nasal passages, intraoral intubation was carried out first working on one side of the mouth and then shifting the tube to work on the other side.

We used the MAS model because it allows safe and efficient treatment of these previously well-selected patients, without the need for the hospital admission. The model makes it possible to shorten the traumatic period for which the patients are removed from their usual environment (family or institution), with a lesser risk of nosocomial infection, and avoiding the complex in-hospital management difficulties these patients pose. The model moreover guarantees the availability of a supporting hospital for the continuation of patient care if needed (26,27). Furthermore, telephone follow-up of the postoperative condition of the patient is made 5 hours after discharge home.

Postoperative complications are not frequent (28,29) though Escamilla-Casal (30) reported a higher rate of complications (bleeding and drowsiness) in a study of children with mental disabilities. In contrast, in our postoperative analysis, over one-half of the patients in both groups showed optimum tolerance and minimum bleeding. Only a small percentage (7%) showed tolerance problems due to nausea, with equal distribution between both groups. With regard to pain following discharge home, the disabled patients showed better scores than the controls. This may have been because surgical aggression was generally less severe in disabled patients than in the controls subjected to oral surgery. Moreover, patients with mental disabilities may have more difficulties in expressing low-intensity pain sensation. In fact, pain intensity was interpreted by the caregivers and reported to the nurse making the telephone call – a circumstance that may produce information bias.

To summarize, it must be taken into account that our study was carried out in a public general hospital in which the different procedures are highly standardized. An interesting observation was that the degree of postoperative wellbeing among the disabled patients was considerably better than in the controls subjected to oral surgery. The ultimate aim of dental treatment under general anesthesia in patients with special needs was to afford basic dental rehabilitation capable of avoiding oral septic foci in a single session and with improved quality outcomes for the patient (14).

## Conclusions

The dental treatment of mentally disabled patients under general anesthesia, based on a MAS model, is safe and affords greater patient comfort than a conventional hospital admission, with a very low incidence of complications. It may be regarded as an adequate management strategy for such uncooperative patients that prove difficult to treat correctly. Patients with severe disorders (ASA III) may be included in this strategy. The anesthesia and surgery times are longer in mentally disabled patients because of the intraoperative management difficulties they pose, but the time to discharge after surgery is no longer than in other types of patients. Lastly, the postoperative patient condition after discharge home is seen to be better than among the controls in surgical procedures of this kind. Further studies should explore how to create a standardized protocol for performing dental treatments for disabled people under general anesthesia.
